# Prophylactic surgery plus hyperthermic intraperitoneal chemotherapy (HIPEC CO2) versus standard surgery for gastric carcinoma at high risk of peritoneal carcinomatosis: short and long-term outcomes (GOETH STUDY)—a collaborative randomized controlled trial by ACOI, FONDAZIONE AIOM, SIC, SICE, and SICO

**DOI:** 10.1186/s13063-022-06880-y

**Published:** 2022-12-01

**Authors:** A. Di Giorgio, C. Gerardi, C. Abatini, G. Melotti, L. Bonavina, V. Torri, F. Santullo, S. Garattini, M. De Luca, Erica Rulli, Eliana Rulli, F. Pacelli, GOETH Investigators

**Affiliations:** 1 Operational Unit of Peritoneum and Retroperitoneum Surgery, Fondazione Policlinico Universitario Agostino Gemelli IRCCS, Largo A. Gemelli, 8, 00168 Rome, Italy; 2 Istituto Di Ricerche Farmacologiche “Mario Negri” IRCCS, Milan, Italy; 3 Associazione Chirurghi Ospedalieri Italiani, ACOI, Rome, Italy; 4 Division of General and Foregut Surgery, Department of Biomedical Sciences for Health, IRCCS Policlinico San Donato and University of Milan, Milan, Italy

**Keywords:** Gastric cancer, Prophylactic surgery, HIPEC CO_2_, Hyperthermic intraperitoneal chemotherapy, Mitomycin, Cisplatin

## Abstract

**Introduction:**

At the time of diagnosis, 15–20% of gastric carcinomas are in stage T4 or T4b. Furthermore, 5–20% of patients undergoing potentially curative surgery suffer from synchronous or metachronous peritoneal metastases. To date, neither surgery nor systemic chemotherapy successfully controls peritoneal dissemination, offering a limited impact on survival. Peritoneal metastases are in fact responsible for death in around 60% of gastric cancer patients.

Several Eastern studies in the past have focused on hyperthermic intraperitoneal chemotherapy (HIPEC) as a prophylactic measure in patients with serosal extension, nodal involvement, and positive peritoneal fluid cytology. Therefore, a new multimodal therapeutic strategy based on aggressive surgery plus new locoregional treatment may prolong survival in this particular clinical scenario.

**Methods:**

This study compares the efficacy of prophylactic surgery (radical gastric resection, appendectomy, resection of the round ligament of the liver, and bilateral adnexectomy) plus hybrid CO2 HIPEC system versus standard surgery in patients with T3-T4 N0-N + gastric adenocarcinoma. Patients will be randomly assigned (1:1 ratio) to the experimental arm or standard surgery. The primary endpoint is to establish the difference in disease-free survival between the groups. The secondary objective is to compare the safety and tolerability of prophylactic surgery plus HIPEC CO_2_ versus standard surgery.

**Discussion:**

Considering the poor prognosis of patients with peritoneal dissemination from gastric cancer, a prophylactic strategy to prevent peritoneal metastases may be beneficial. In patients with gastric cancer at high risk of peritoneal carcinomatosis, we propose aggressive surgical treatment with radical gastrectomy, removal of organs at risk of harbouring tumour cells, and HIPEC.

**Trial registration:**

ClinicalTrials.gov NCT03917173. Registered on 16 April 2019.

Protocol version: v1, March 27, 2019.

Protocol number: IRFMN-GCC-7813.

EudraCT number: 2019–001478-27.

**Supplementary Information:**

The online version contains supplementary material available at 10.1186/s13063-022-06880-y.

## World Health Organization Trial Registration Data Set information

1. Primary registry and trial identifying number: NCT03917173 (http://clinicaltrials.gov)

2. Date of registration in primary registry: April 26

2019

3. Secondary identifying numbers: n/a

4. Source(s) of monetary or material support: unconditional grant from ACTA group, Naples, Italy

5. Primary sponsor: Associazione Chirurghi Ospedalieri Italiani (ACOI)

6. Secondary sponsor(s): n/a

7. Contact for public queries: Andrea Di Giorgio, andrea.digiorgio@policlinicogemelli.it

8. Contact for scientific queries: Carlo Abatini, carlo.abatini@guest.policlinicogemelli.it

9. Public title: Prophylactic surgery plus hyperthermic intraperitoneal chemotherapy (HIPEC CO2) versus standard surgery in gastric carcinoma at high risk of peritoneal carcinomatosis: short and long-term outcomes (GOETH STUDY)—a collaborative randomized controlled trial by ACOI, FONDAZIONE AIOM, SIC, SICE, and SICO

10. Scientific title: Prophylactic surgery plus hyperthermic intraperitoneal chemotherapy (HIPEC CO2) versus standard surgery in gastric carcinoma at high risk of peritoneal carcinomatosis: short and long-term outcomes (GOETH STUDY)—a collaborative randomized controlled trial by ACOI, FONDAZIONE AIOM, SIC, SICE, and SICO

11. Countries of recruitment: Italy

12. Health condition(s) or problem(s) studied: gastric carcinoma at high risk of peritoneal carcinomatosis

13. Intervention(s):

- Experimental: prophylactic surgery plus HIPEC CO_2_ with mitomycin and cisplatin
- Comparator: standard surgery

14. Key inclusion and exclusion criteria: see the “ Method and analysis” section

15. Study type: randomized, multicentre, controlled trial with two arms (1:1 allocation ratio)

16. Date of first enrollment: June 1, 2020

17. Target sample size: 240 patients

18. Recruitment status: recruiting

19. Primary outcome(s): disease-free survival

20. Key secondary outcomes: overall survival, local recurrence-free survival, post-surgery complications, morbidity, duration of surgery, number of patients receiving the adjuvant chemotherapy, length of hospitalization, mortality 30 and 90 days from surgery

## Strengths and limitations of this trial

### Strengths

- Rationale for CO_2_ infusion that generates intra-abdominal turbulence to overcome the drug distribution issues of the closed approach
- Randomized controlled trial with a novel HIPEC system with simultaneous use of the HIPEC technique since all participating centres will follow the same HIPEC protocol
- No delay between surgery and HIPEC

### Limitations

- Possible delay in starting adjuvant treatment in the experimental arm because of the added chemotherapy
- No preoperative stratification based on tumour pathology or mutational profile
- Different perioperative systemic chemotherapy regimens allowed
- Difficulty in separately analysing the effect of extended surgery from HIPEC in experimental group

## Background

Gastric cancer is the fifth most common form of neoplasm worldwide and third for mortality [1].

At diagnosis, 15–20% of gastric carcinomas are in stage T4 or T4b [2]. Synchronous or metachronous peritoneal metastases are very common in patients with locally advanced gastric carcinomas, affecting 5–20% of those undergoing potentially curative surgery. If serosal surface invasion is a logical risk factor for carcinomatosis, also T3 cancer has a considerable risk of peritoneal metastases, especially for Lauren diffuse type [3–5].

For diffuse adenocarcinomas, the incidence ranges between 30 and 60% and can be as high as 80% if peritoneal fluid cytology tests positive [3, 5]. Moreover, after R0 gastrectomy surgery, the peritoneal recurrence rate is about 30%.

To date, neither surgery nor systemic chemotherapy ensures satisfactory control of peritoneal dissemination and has no significant impact on survival [6]. Systemic chemotherapy has a limited effect, with an average survival of 7 to 12 months [7–10], and peritoneal metastases are the cause of death in around 60% [11].

So far, the literature seems to point towards perioperative systemic chemotherapy as a first-intention option in potentially resectable, locally advanced gastric carcinoma. Perioperative chemotherapy has an advantage over surgery alone in terms of survival, with reductions in the relative risk (19%) and absolute risk (9%) of recurrence of the disease. Perioperative poly-chemotherapy has also given a survival advantage over adjuvant chemotherapy alone [12, 13].

Hyperthermic intraperitoneal chemotherapy (HIPEC) is suggested as a treatment integrating cytoreductive surgery for carcinomatosis and as a precautionary strategy in locally advanced gastric carcinoma at risk of recurrence, with the aim of improving overall survival and reducing peritoneal recurrence [14]. The theoretical advantages of HIPEC consist in administering large quantities of antiblastic drugs to the abdominal cavity, reducing their systemic toxicity, and exploiting the synergistic effect of hyperthermia which contributes to antitumoural efficacy in several ways [15, 16].

Several Asian trials have focused on HIPEC as a prophylactic measure in patients with serosal extension, nodal involvement, and positive peritoneal fluid cytology. Most of them were conducted between 1988 and 2001, using mitomycin and cisplatin for intraperitoneal chemotherapy and with variable HIPEC temperatures and flow rates. No adjuvant or neoadjuvant systemic chemotherapy was used in these studies and the 5-year overall survival (OS) ranges from 42 to 66% in the experimental groups. These pioneering Asian experiences had encouraging oncological outcomes, theoretically supporting the use of HIPEC for the prevention of peritoneal recurrence in gastric cancer [17–22].

These results are also confirmed by two meta-analyses: Coccolini et al. studied 20 randomized clinical trials on surgery with intraperitoneal chemotherapy in patients with advanced gastric cancer; 2145 patients were included: 1152 of them treated with surgery plus intraperitoneal chemotherapy and 993 surgery alone. The meta-analysis showed that surgery with intraperitoneal chemotherapy reduced overall mortality at 1, 2, and 3 years; mortality at 2 and 3 years in patients with locoregional lymph node metastasis; mortality at 1 and 2 years in patients with serosal involvement; and the rates of hematogenous metastases and peritoneal recurrence [5]. Feingold et al. in a systematic review with 2029 treated patients reported that intraoperative chemotherapy in patients at high risk of peritoneal carcinomatosis from gastric cancer reduced mortality at 5 years [14].

These considerations raise the question of whether the results apply to the western population. The lack of solid evidence has led national and international clinical guidelines not to support the use of adjuvant HIPEC outside a clinical trial.

The GOETH study was designed to address this knowledge gap.

### Impact of the COVID-19 pandemic on prevention, cancer detection, and treatments

A recent WHO survey showed that 75% of countries reported a considerable degree of noncommunicable disruption of services due to the COVID-19 pandemic. This was consistent across all regions and income groups. The most common reasons for service disruptions were cancellation of elective care, lack of transport due to lockdowns, staff shortages, and closure of hospital services. Globally, 2.3 million cancer surgeries were cancelled or postponed during the peak 12-week period of COVID-19. One main reason for these disruptions of services was the closure of population-level screening programmes and lockdowns, hindering access to health facilities [23].

A paper from Nature points out that modelling the effect of COVID-19 on cancer screening and treatment for breast and colorectal cancer (which together account for about one-sixth of all cancer deaths) over the next decade will see almost 10,000 excess deaths from these cancers. This is a roughly 1% increase in deaths from these tumours during a period when one could expect almost 1,000,000 deaths from the two diseases. According to this predictive model, the number of excess deaths per year should peak in the next year or two [24].

Major oncology scientific societies have therefore recommended the use of telemedicine and boosting local medicine. At the European level, telemedicine has been recommended for follow-up visits and monitoring oral drug-based therapy [25].

## Method and analysis

### Hypothesis

In patients with gastric cancer at high risk of peritoneal carcinomatosis (PC), primary radical tumour resection with D2 lymphadenectomy, combined with a more aggressive surgical approach and prophylactic HIPEC, should reduce peritoneal recurrence.

### Primary objective

The primary objective of the study is to compare the efficacy of prophylactic surgery with HIPEC CO2 versus standard surgery in terms of disease-free survival (DFS) in patients with gastric carcinoma (GC) at high risk of developing peritoneal carcinomatosis.

### Secondary objectives

- To compare the experimental treatment (prophylactic surgery plus mitomycin- and cisplatin-based HIPEC CO_2_) versus standard treatment on local recurrence-free survival (LRFS) and OS
- To assess the safety (treatment-related morbidity and mortality) of this experimental approach
- To assess the number of patients performing the adjuvant treatment

### Study design

This is a phase III, randomized, multicentre, superiority trial in patients with gastric carcinoma, at high risk of peritoneal carcinomatosis. Patients may have had neoadjuvant chemotherapy according to clinical practice, or direct surgery. If, after diagnostic exams, patients are eligible for the trial and resection of the tumour is total during surgery, patients will be randomly assigned (1:1 ratio) not more than 24 h before surgery to prophylactic surgery plus HIPEC CO_2_ (arm A) or to standard surgery (arm B). The primary objective is to compare the efficacy of prophylactic surgery (radical gastric, appendectomy, resection of the round ligament of the liver, and bilateral adnexectomy) plus HIPEC CO_2_ versus standard surgery in terms of DFS. The secondary objective is to compare the safety profile and tolerability of prophylactic surgery plus HIPEC CO_2_ versus standard surgery.

### Participants

The target population comprises patients with gastric carcinoma, at high risk of peritoneal carcinomatosis. The inclusion and exclusion criteria are reported below.

#### Inclusion criteria

1. Patients with histologically documented gastric carcinoma (diffuse/intestinal histotype) eligible for R0 with (a) presurgical stage T3-T4 N0-N + primary tumour (TNM 8th), (b) urgent presentation: perforation without purulent generalized peritonitis, and (c) positive cytology of peritoneal fluid (if previously obtained)
2. Age ≥ 18 years and ≤ 75 years
3. Written informed consent

#### Exclusion criteria

1. Gastroesophageal junction (GEJ) cancer
2. Distant metastatic disease (even if limited and completely resected)
3. Peritoneal carcinomatosis
4. History of tumour diagnosed in the 3 years before entering the study, except for topical and healed pathologies that do not need further treatment (e.g. non-melanoma skin carcinomas, superficial bladder carcinomas, or in situ carcinoma of the breast or cervix)
5. Psychological, family, or social conditions which may negatively affect the treatment and follow-up protocol
6. Poor general condition (ECOG > 2)
7. Impaired cardiac function (history of congestive heart failure or FE < 40%). Clinically significant cardiovascular disease: cerebral vascular accident/stroke (< 6 months prior to enrolment), unstable angina, congestive heart failure (New York Heart Association Classification Class > II), or serious uncontrolled cardiac arrhythmia requiring medication
8. Impaired renal function (creatinine > 1.5 upper limit of normal or creatinine clearance < 60 mL/min)
9. Impaired hepatic function (AST, ALT > 2.5 upper limit of normal, bilirubin > 1.5 upper limit of normal)
10. Impaired haematopoietic function (leucocytes < 4000/mm^3^, neutrophils < 1500/mm^3^, platelets < 100,000/mm.^3^)
11. Impaired pulmonary function (presence of COPD or other pulmonary restrictive conditions with FEV1 < 50% or DLCO < 40% of normal age value)
12. History or presence of other disease, metabolic dysfunction, or clinical laboratory finding giving reasonable suspicion of a disease or condition that contraindicates the use of HIPEC or chemotherapy or patient at high risk from treatment complications
13. Pregnancy
14. Krukenberg tumour
15. Refusal to join the study

### Randomization

Patients will be randomized no more than 24 h before surgery if total resection of the tumour is intended, and a stratification procedure based on centre and neoadjuvant chemotherapy will be used. Patients will be randomized in a 1:1 ratio. The randomization lists were prepared by the study statistician using the SAS system (version 9.4).

### Treatment regimen

Patients assigned to arm A will receive prophylactic surgery and HIPEC CO_2_ with cisplatin and mitomycin in addition to primary tumour resection. Patients randomized to standard surgery (arm B) will be operated to clinical practice, without HIPEC CO_2_.

During surgery, surgeons will assess the presence of peritoneal carcinomatosis, and if it is found, patients randomized to arm A will not receive HIPEC and will be operated to surgery according to clinical practice.

### Surgery

Diagnostic laparoscopy is suggested. Before surgical resection, peritoneal washing will be done for a definitive cytological examination. Both the laparotomic and laparoscopic surgical approaches are allowed according to clinical practice. In the experimental arm, the prophylactic surgery will include radical gastrectomy with D2 lymphadenectomy and omentectomy, resection of the round ligament of the liver, bilateral adnexectomy, and appendectomy. For women of child-bearing age, bilateral adnexectomy should be discussed. In the comparator arm, radical standard surgery will be done (radical gastrectomy with D2 lymphadenectomy). For both arms, if necessary, multivisceral resection could be done to achieve R0.

### Hyperthermic intraperitoneal chemotherapy (HIPEC) procedure

In experimental arm A, patients will undergo HIPEC. We will use a closed-abdomen HIPEC technique with CO_2_ agitation, using a specific CE-marked device with multi-perforated catheters, two placed in the upper abdomen for the chemotherapy infusion and the other two in the lower abdomen for fluid aspiration and CO_2_ infusion. Chemotherapy with the perfusion solution at 42 °C and CO_2_ flowed into the abdominal cavity; turbulent flow was created to improve drug distribution. HIPEC may be done after laparoscopic or laparotomic primary tumour resection without interference with the standard surgical techniques.

The HIPEC CO_2_ regimen will be as reported below: mitomycin 15 mg/mq and cisplatin 75 mg/mq both in physiologic solution 0.9%.

The recommended temperature for HIPEC treatment is 42 °C for 60 min of perfusion.

Sodium thiosulfate will be administered to prevent nephrotoxicity induced by cisplatin as follows: sodium thiosulfate 9 g/mq bolus and then sodium thiosulfate 1.2 g/mq/h with continuous infusion for 6 h. Adequate preoperative and postoperative iv hydration is necessary.

### Perioperative and adjuvant chemotherapy

Clinically staged T3-4 N0-1 M0 patients should be considered for a perioperative approach.

Neoadjuvant treatment consists of 3 months of chemotherapy followed, after surgery, by another 3 months of chemotherapy according to the same regimen used preoperatively. One can choose between epirubicin, cisplatin, fluorouracil/capecitabine (ECF/ECX) or oxaliplatin, capecitabine (XELOX) or oxaliplatin, fluorouracil/leucovorin (FOLFOX-4) or fluorouracil, leucovorin, oxaliplatin, docetaxel (FLOT). For patients who receive FLOT before surgery, chemotherapy could be changed after surgery in case of toxicity or because of a medical decision. Patients not receiving neoadjuvant chemotherapy will be given chemotherapy for 6 months in the adjuvant setting (consider ECF/ECX or FOLFOX/XELOX regimen). Adjuvant treatment should start within 8–12 weeks from surgery.

### Disease assessment

Before randomization, complete blood count (CBC), blood chemistry (glucose, sodium, potassium, chloride, blood urea nitrogen (BUN), creatinine, alanine aminotransferase (ALT), alkaline phosphatase (ALP), gamma glutamyl transpeptidase (GGT), aspartate transaminase (AST), total and direct bilirubin, albumin, and total protein), coagulation tests (prothrombin time (PT), activated partial thromboplastin time (aPTT), INR), tumour markers (CEA, CA 19.9), B-HCG test, and electrocardiogram test will be scheduled according to clinical practice. No biological material will be collected and stored. Thoraco-abdominal computed tomography is mandatory before surgery and evaluation with endoscopic ultrasound (EUS) is suggested. Moreover, information about patients’ anamnesis and primary tumour characteristics will be collected.

A computed tomography scan (CT scan) should be done 6 weeks after surgery and adjuvant treatment should start within 8 weeks from surgery. Histological examination after surgery is required in order to confirm clinical and radiological findings before surgery according to TNM.

### Study endpoints

#### Efficacy

The primary efficacy endpoint is disease-free survival. DFS is defined as the time from randomization to the date of first local relapse, distant relapse, peritoneal carcinomatosis, or death for any cause, whichever comes first. Patients alive and without relapse will be censored at their last disease evaluation.

The secondary efficacy endpoints are local recurrence-free survival (LRFS) and overall survival.

LRFS is defined as the time from randomization to the date of first local relapse, peritoneal carcinomatosis, or death for any cause, whichever comes first. OS is defined as the time from randomization to death for any cause.

#### Safety

The safety endpoints will be mortality 30 and 90 days from surgery, morbidity during and after surgery (graded according to the NCI-CTAE version 4.03 for AE related to chemotherapy and according to Clavien-Dindo for surgery complications) (34, 41–42), the number of post-surgery complications, the duration of surgery, the length of hospitalization, and the number of patients receiving the adjuvant chemotherapy.

### Sample size

The sample size calculation considers the amount (15%) of patients with peritoneal carcinomatosis undetected by CT scan and discovered only during the surgical procedure. Since the primary endpoint will be analysed according to the ITT approach, no further loss of patients was hypothesized.

Patients will be randomized no more than 24 h before surgery if total resection of the tumour is intended, and a stratification procedure based on the centre and neoadjuvant chemotherapy will be used. Patients will be randomized in a 1:1 ratio. The randomization lists were prepared by the study statistician using the SAS system (version 9.4). Regarding the sample size, the calculation was based on the following assumption:

- Median PFS in the control arm: 1 year
- Constant hazard rate
- Relative reduction in hazard rate: 33%
- Alfa error: 4.9% 2 sides
- Power: 80%

According to STATA Module ART (ART: Stata module to compute sample size and power for complex randomised trial designs with binary or time-to-event outcomes Abdel Babiker (a.babiker@ucl.ac.uk), Friederike Maria-Sophie Barthel (sophie@fm-sbarthel.de), Babak Choodari-Oskooei (b.choodari-oskooei@ucl.ac.uk), Patrick Royston (j.royston@ucl.ac.uk), Ella Marley-Zagar (e.marley-zagar@ucl.ac.uk), and Ian White (ian.white@ucl.ac.uk)), the number of required events is 200. Since the study is event driven, it will be stopped when this required number will be reached. In order to estimate the total sample size, we need to assume the duration of recruitment, the total study duration, and the drop-out rate.

If uniform accrual of 3 years and overall observation time of 6 years are assumed, 220 patients are required. This number has been furtherly increased to 240 to allow for a slower than expected accrual and a consequent longer accrual time, maintaining 6 years of total time. To consider also an overall dropout rate of approximately 15%, 282 patients need to be randomized.

### Statistical analysis

Efficacy will be analysed on the modified intention to treat (mITT) population, including all patients randomized, without major violations of eligibility criteria and no evidence of peritoneal carcinomatosis (detected during surgery).

DFS and OS will be described with the Kaplan–Meier method. Differences between arms will be tested by the log-rank test and by univariate and multivariate Cox’s models, including stratification variables and other clinical-biological features as covariates. LRFS will be described with a cumulative incidence function and will be analysed with a Grey test to take account of the competing risks.

An interim analysis of efficacy will be done when half of the events have been observed. The conservative Haybittle-Peto boundary will be used as stopping guidance to ensure final analysis at the significance level of 0.049.

No imputation of missing data will be done.

### Data collection, management, and analysis

Data will be collected using an electronic case report form (CRF). A data timing plan and data validation plan, developed by the statisticians and data managers of the coordinating centre, will be used to request data input (RID) in the electronic CRF and to check the data entered by data clarification forms (DCF). The sponsor maintains confidentiality standards by assigning a unique patient identification number to code.

### Quality assurance

Each investigator will be responsible for ensuring data quality, as planned in the Data Validation Plan. Each item of information in the electronic CRF will be systematically checked for consistency, completeness, or incongruity by the Data Coordinating Center, which will issue DCFs in case of inconsistent data. Local quality control will be provided by the coordinating centre, which will be responsible for monitoring all the centres.

### Monitoring the trial

During the trial, a sponsor’s representative will have regular contact with the study site, including visits to provide information and support for the investigator(s), confirm that the investigational team is adhering to the protocol, that data are being accurately and timely recorded in the eCRFs; to verify source data (comparison of the data in the eCRFs with the patient’s medical records at the hospital or practice, and other records relevant to the study) including verification of informed consent.
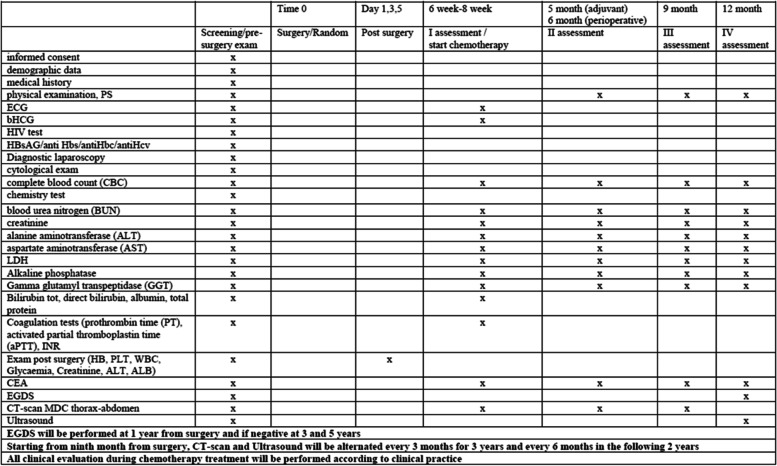


Authorized representatives of a regulatory authority or Ethics Committee may perform audits or inspections at the study centres, including source data verification.

### Trial management

#### Administrative structure

The coordinating centre is the Policlinico Universitario Agostino Gemelli IRCCS, Roma.

The sponsor is “Associazione Chirurghi Ospedalieri Italiani (ACOI)”, which has delegated the Mario Negri Institute for Pharmacological Research as the Data Coordinating Center for oversight of clinical operations, data management support, and clinical monitoring.

About 60 experimental centres are expected to participate. These centres have been selected based on the report and recommendation of the Italian National Agency for Regional Healthcare Services (AGENAS) with at least 30 surgeries for gastric disease per year. It will also be possible to include centres from other countries.

#### Independent data monitoring committee (IDMC)

An independent data monitoring committee (IDMC) comprising three international experts (one oncologist, one surgeon, and one statistician), not involved in the trial and with no conflict of interest with respect to the results, will monitor the progress of the trial from the ethical and scientific viewpoints. The IDMC will review the interim efficacy analysis and the safety reports in order to monitor toxicity. Based on this, the IDMC will provide recommendations to the study sponsor and the Steering Committee (SC).

#### Safety reporting

The collection, assessment, and presentation of safety reports will be carried out in accordance with the detailed guidance on the collection, verification, and presentation of adverse event/reaction reports arising from clinical trials on medicinal products for human use (European Commission guidance on adverse reaction reporting (ENTR/CT3)).

Patients will be carefully monitored for any AE occurring during the trial conduct. Such monitoring also includes clinical laboratory tests. AEs will be assessed in terms of their seriousness, severity, and causal relation to the study treatment. Safety reporting to study investigators, ECs, and competent authorities will then follow in accordance with the results of such assessment.

Severity and seriousness will be independently assessed for each AE and recorded on the e-CRF.

### Ethics approval, consent to participate, and dissemination

The trial will be conducted in accordance with the ethical principles set out in the Declaration of Helsinki and are consistent with ICH/Good Clinical Practice and regulatory requirements for participant data protection.

Prior to entering the study, patients will be given key information about the trial, verbally and in a written consent form. Patients are notified that they are free to withdraw from the trial at any time.

The use of participant data and biological samples is not intended for further ancillary studies.

The study has been approved by the Ethics Committee of the Università Cattolica, Policlinico Agostino Gemelli IRCCS (protocol IRFMN-GCC- 7813), Rome, and has been approved or is under evaluation by the Ethics Committees of all the participating centres. Any substantial amendment made to the protocol by the coordinating investigator is submitted to the local ethics committee and health authorities for approval, prior to implementation.

According to local and international regulations, the trial results are the property of the sponsor who will share them with all participating investigators. There is a commitment to post trial results in a public register 1 year after the trial is completed and to publish results, irrespective of the findings, in a peer-reviewed journal.

The sponsor maintains confidentiality standards by coding each patient enrolled in the study through assignment of a unique patient identification number.

Records and documents pertaining to the conduct of this study, including eCRFs, ICFs, and investigator site files (ISFs) must be retained by the principal investigator for at least 15 years after completion or discontinuation of the study or for the length of time required by relevant national or local health authorities, whichever is longer. After that period of time, the documents may be destroyed, participant to local regulations.

The sponsor of the study (ACOI) agrees with Istituto di Ricerche Farmacologiche Mario Negri IRCCS to take out adequate clinical insurance to cover its obligations, including but not limited to providing compensation to patients in the study suffering injury of death or loss caused by the administration of drugs or any clinical intervention or procedure in accordance with the relevant protocol and all legal requirements. All patients participating in this clinical trial will therefore be covered by a civil liability policy in accordance with the DM 14–07-2009.

Results derived from the trial are the property of the sponsor which shares them with all participating investigators and regulatory authorities. We plan to share the results with the scientific community and national gastric cancer patient associations.

Publications will be decided by the SC. Authors to be reported in the front page will be selected on the basis of the specific contribution or the number of enrolled patients and/or on the consistency, completeness, and accuracy of the data.

### Standard for protocol publication

This clinical trial protocol follows the Standard Protocol Items: Recommendations for Interventional Trials (SPIRIT) guidelines. The trial is registered on clinicaltrial.gov (NCT039171730).

### Patient and public involvement

Patients and/or the public were not involved in the design, or conduct, or reporting, or dissemination plans of this research.

## Discussion

The original study design was based on intraoperative randomization of the patients. However, organizational difficulties arose during the study conduction in applying the intraoperative randomization, amplified by the COVID-19 pandemic, and in June 2021, an amendment was planned to guarantee the feasibility of the trial. The amendment modified the protocol, moving randomization from intraoperative to 24 h before surgery. In the previous design, patients with peritoneal carcinosis detected during surgery did not access the randomized part of the study, allowing an ITT approach.

By placing the randomization before the intervention, patients with peritoneal carcinosis diagnosed during surgery and not identified at the pre-surgery diagnostic level will be included and randomized. These patients will not receive the experimental treatment and are not part of the target population defined by the eligibility criteria. In this group (assuming 10–20% of the sample), the difference between experimental and control treatment is expected to be null, with a consequent dilution of the total effect, i.e. the hazard ratio (HR) resulting from the primary analysis will approach 1 with respect to the hypothesized effect. In addition, the results would be difficult to interpret due to the population included. Therefore, in the amended protocol, a modified ITT was applied, excluding patients with peritoneal carcinosis diagnosed during surgery to obtain results on the target population without losing the generalizability.

Finally, the number of randomized patients was increased from 240 to 282, to take account of the expected number of patients (around 15%) with peritoneal carcinosis detected during surgery and excluded from the primary analysis.

Current, national, and international guidelines do not include HIPEC for advanced or metastatic gastrointestinal cancer mainly because of the lack of large clinical trials in western countries, and any clinical benefit of adjuvant HIPEC for gastric cancer has yet to be demonstrated. The GOETH trial has been designed to address this gap and test the clinical advantage of a surgical and chemotherapeutic prophylactic strategy to prevent recurrence. Therefore, we set DFS as the primary endpoint.

The HIPEC regimen of the present trial is based on cisplatin and mitomycin. This was supported by the results of past Asian prophylactic studies and the GYMSSA trial, which explored a similar HIPEC regimen after cytoreductive surgery, with curative intent [26].

There is no standardized HIPEC protocol in clinical practice nor has a consensus been reached. The use of a single HIPEC technique in all centres in our trial appears to be one of the most important strengths of the protocol. The closed CO2 recirculation offers adequate peritoneal surface drug exposure, stability, and homogeneity of the intra-abdominal temperature and should overcome the shortcomings inherent to the closed technique. Both the laparoscopic and laparotomic surgical approaches can be safely used using this device. Another theoretical feature of this new HIPEC system, not been yet demonstrated, could be increased drug penetration through the mesothelial surface due to higher abdominal pressure. No deleterious impact on blood or hemodynamic parameters was found in an animal model and our own clinical experience has confirmed that the technique is safe and feasible, with good perioperative outcomes [27–31].

The experimental arm surgery comprises prophylactic surgical excision of organs at risk of peritoneal metastases, to prevent microscopic peritoneal dissemination from the spread dynamics of peritoneal fluid. This strategy was already proposed by Sammartino et al. for colorectal cancer with promising results and no increasing morbidity [32, 33].

Ovarian metastases are frequent in patients with gastric cancer, and this is the first most frequent site of origin. In a series of 2515 patients from the G.I.R.C.G. (Gruppo Italiano Ricerca Cancro Gastric), 30 presented with synchronous Krukenberg tumour and 33 developed metachronous ovarian metastases. Survival in the metachronous group was better than in the synchronous group [34]. The introduction of another variable in the experimental treatment brings a confounding bias when assessing the role of HIPEC. However, the removal of organs at high risk of PM reduces the risk of primary ovarian or appendiceal cancer, removes microscopic synchronous metastases, and prevents metachronous relapse.

In our trial, several neoadjuvant regimens are accepted and patients not receiving any preoperative chemotherapy can be enrolled. On this point, we opted to widen the indications, also considering the heterogeneity in current clinical practice in Italy. To minimize the bias introduced, the neoadjuvant chemotherapy regimen is considered a stratification variable for randomization.

All patients will receive adjuvant chemotherapy, unless there are clinical contraindications, this being current clinical practice in Italy.

Evidence from Western countries currently available on adjuvant HIPEC for gastric cancer is recent and under development. Reutovich et al. recently published the results of a Belarus randomized trial including 154 advanced gastric cancer patients treated with surgery or surgery + HIPEC with cisplatin 50 mg/m^2^ and doxorubicin 50 mg/m^2^. No other adjuvant treatment was scheduled. The trial was powered on progression-free survival (PFS) and demonstrated that early peritoneal recurrence was reduced, showing a significant advantage on 4-year PFS (*p* < 0.001). However, this did not translate into a significant survival benefit. The peritoneum may not be the only site of metastases and patients did not receive any adjuvant systemic chemotherapy [35].

The risks for adjuvant HIPEC in a prophylactic setting remain controversial with significant heterogeneity in published literature. The types of adverse events are heterogeneous and attributable to the effect of chemotherapy and hyperthermia: bone marrow suppression, liver and kidney disfunction, infectious, intestinal fistula, and anastomotic leak. The systematic review of Feingold et al. and the meta-analysis of Sun et al. demonstrates that intraperitoneal chemotherapy has good oncological outcomes and does not increase complications [14, 36].

Conversely, the meta-analysis by Desiderio et al. found a higher rate of adverse events related to HIPEC administration, especially renal disfunction [37].

Various randomized clinical trials in Europe are enrolling in the prophylactic setting, investigating different drugs, perfusion techniques, and systemic treatments. The GASTRICHIP (NCT01882933) is a well-known actively recruiting European phase III trial that shares some characteristics with our study, mainly inclusion criteria. Differences are the extension of surgery in the experimental group (radical gastrectomy with D1-D2 lymphadenectomy), HIPEC technique (open or closed are permitted), and peritoneal drugs (oxaliplatin with intravenous 5-FU and calcium levofolinate induction). The primary endpoint is OS and the secondary endpoints are recurrence-free survival, morbidity, and quality of life.

The CHIMERA trial (NCT04597294) is a Polish phase III trial, not yet recruiting, testing the efficacy of perioperative FLOT4 chemotherapy in combination with HIPEC in 600 patients with advanced gastric cancer at high risk of peritoneal metastases. In the experimental arm, the HIPEC schedule is based on irinotecan 300 mg/m^2^ infused for 45 min at 42°. The primary endpoint is the rate of peritoneal recurrences at 6 months from randomization.

The PREVENT trial (NCT04447352) is a German recruiting phase III trial evaluating the efficacy and safety of perioperative FLOT chemotherapy plus intraoperative cisplatin-based HIPEC (75 mg/m^2^) versus FLOT chemotherapy alone in patients with resectable locally advanced adenocarcinoma of the stomach and gastroesophageal junction (type II/III). In this study, the primary outcome is the comparison of progression-/disease-free survival (PFS/DFS) between arms.

Our study represents a novelty in this evolving context with some specific characteristics which, if proved, could improve the management of advanced gastric cancer.

## Conclusion

The GOETH trial is the only one where prophylactic surgery and a new HIPEC technique with CO_2_ agitation are employed in patients with advanced gastric cancer, treated with an up-to-date multimodal strategy.

Considering the poor prognosis of peritoneal carcinomatosis from gastric cancer, maximum efforts are needed to identify and adequately treat patients with clinical and pathological risk factors.

In this randomized trial, an aggressive surgical strategy comprising radical gastric resection, removal of organs at risk of harbouring tumour cells, and HIPEC, is compared against standard surgery for advanced gastric cancer.

## Supplementary Information

**Additional file 1.**

## Data Availability

Systematic individual patient data sharing is not intended, but all requests for the trial’s data, full protocol, and statistical analysis plan will be considered by the Steering Committee upon request.
