# Supplementary material for: Prophylactic surgery plus hyperthermic intraperitoneal chemotherapy (HIPEC CO2) versus standard surgery for gastric carcinoma at high risk of peritoneal carcinomatosis: short and long-term outcomes (GOETH STUDY)—a collaborative randomized controlled trial by ACOI, FONDAZIONE AIOM, SIC, SICE, and SICO
Source: Trials. 2022 Dec 1;23:969. doi: 10.1186/s13063-022-06880-y (PMC9714394; doi:10.1186/s13063-022-06880-y)
Supplement: Supplementary file 1 — Additional file 1. [file 13063_2022_6880_MOESM1_ESM.pdf]

17. PUBLICATION POLICY

Results derived from the trial are property of the Sponsor which shares them with all participating investigators.
Every publication of the study results will be written on the basis of the analyses performed by the coordinating data center and approved by the SC. Publications will be decided by the SC. Authors to be reported in the front page will be selected on the basis of the specific contribution or the number of enrolled patients and/or on the consistency, completeness and accuracy of the data. Furthermore, the specification “on behalf of GOETH Study Group” will be added. The name list of each article will include: For each experimental centre at least one investigator; For the Coordinator the responsible, the statistician, the statistician in charge of interim analysis, the data manager, the responsible of informatics, the local monitors and the responsible of safety desk; - SC members; - One delegate for each scientific society involved; Furthermore, all manuscripts will include an appropriate acknowledgement section, mentioning all persons who have made substantial contributions to the work but who are not authors and sources of funding and support. Rules for abstract presentation will be the same as for extended papers. Commitment to post trial results in a public register one year after the trial is completed, i.e. last follow up of the last patient for the primary outcome and to publish results irrespective of findings.
Rules for abstract presentation will be the same as for extended papers.

17.1. Clinical Study Report

At the end of the study, clinical study report will be written and distributed to all investigators and regulatory authorities
